# Mr. Bartley, on Artificial Musk in Hooping Cough

**Published:** 1801-02

**Authors:** O. W. Bartley

**Affiliations:** Surgeon. Bradford


### *To the Editors of the Medical and Phyfical Journal.*

GENTLEMEN,

AT the conclufion of a paper honoured with infertion in your valuable Mifcellany (No. XIX.) about three months fince, I fignified my intention of treating more largely on the benefit which is derived from the exhibition of the Artificial Mufk in Hooping Cough, as well as in cafes of extreme debility. Leifure now permitting me to make a few obfervations on the fubject I, without further apology, fubmit them to your notice.

It would be unneceffary to make a full and tedious explanation of thofe cafes of hooping cough relieved by the mufk; it will be fufficient to fay, that I have adminiftered it in feveral, and with conftant and uniform good fuccefs. The urgent fymptoms are fpeedily mitigated by this powerful remedy, and the difeafe is quickly fubdued, as it were, by a fpecific action, unindebted to the aid of any other medicine, I never having preceded its exhibition, even with an emetic: Doubtlefsly, others who have adopted its ufe, muft with me have had the fatisfaction of noticing its good effects. I think it neceffary to obferve, that I have conftantly introduced the medicine in form of a tincture, prepared as I have already directed in my laft paper: This has, I think, the advantage of a more fpeedy efficacy, as being more diffufive, and is lefs likely to offend the ftomach, than when taken in fubftance or in an emulfion, for I have obferved the latter frequently to create naufea. Confidering twenty drops of the tincture as a ftandard dofe for an adult, I proportionate the quantity to be taken according to the age, temperament, &c. of my patient. This is, I think, as much as is neceffary for me to fay on this part of the fubject: I fhall, therefore, proceed to tranfcribe a cafe, wherein the tonic and reftorative powers of the mufk are fo prominent, that I think it deferves to be recorded.

Eleanor Sadler, aged about 23 years, a pauper of this parifh, of a light, fanguine temperament, and a habit predifpofed to fcrophula, had been from her earlieft infancy the unhappy victim of difeafe. When very young, the fmall-pox deprived her of the fight of one eye, and greatly endangered the other. She thus continued almoft in a ftate of blindnefs, until the age when catamenia ufually begin to appear, at which time the mifery of her condition was much aggravated, as every method taken within her power, for the promotion of the fecretion, proved ineffectual. She was fhortly afterwards affected with acute pain throughout the whole epigaftric region, attended with a dyfpnœa fo violent, as almoft to preclude the poffibility of the leaft motion or exertion without endangering fuffocation. By applying to the parifh furgeon for relief, fhe obtained a temporary one by venæfection; but, although this was frequently repeated, refpiration ftill continued fo laborious, and the pain fo troublefome, that fhe could not in any degree contribute towards providing a fcanty pittance for her fubfiftence. She continued in this deplorable condition for many years, notwithftanding Mr. Hooper, (the furgeon before alluded to) with the greateft humanity and attention, endeavoured by every mean within his power to *mitigate* her fufferings, having relinquifhed the hope of entirely *removing* them. He at length advifed her to to go to the Briftol Infirmary, and procured a recommendation for her. From thence fhe was foon difmiffed as incurable. During her ftay there, fetons were repeatedly made in her temple, to remove a pain fhe felt in her remaining eye, the anguifh of which was almoft infupportable, there conftantly exuding from it an ichorous corrofive difcharge, except when removed by an artificial drain. Soon after her return to this place, another perfon was nominated to the office of parifh furgeon, who frankly told her, he could do nothing to relieve her, nor would he attempt it. Thus, deferted by the only affiftance her extreme poverty could claim, fhe ventured to intreat my attention to her cafe: I complied with her requeft, and examined her eye, but could difcover no external inflammation, either in the tunica conjunctiva or elfewhere, nor any appearance of caligo; a thin acrid fluid conftantly diftilled in confiderable quantities from under the palpebræ, but I could not difcover its fource. I had recourfe to (as before practifed) the feton in the adjoining temple, and frequently ordered the ufe of collyria with opium, camphor, &c. but the relief thefe afforded were very tranfient. I alfo tried every method I could fuggeft to remove the dyfpnœa, which every day became more alarming; I introduced at different times almoft the whole tribe of demulcents, tonics, alteratives, chalybeates, &c. but all without any permanent advantage. I became at length almoft weary of the coft and trouble I continually experienced on her account, without having in profpect the fatisfaction of eventually finding my pains requited by her recovery. When, in May laft, I determined, as a "derniere refort," to exhibit the artificial mufk, though I had little or no hope of its being attended with fuccefs. I gave her fome of the tincture, and directed her to take twenty or thirty drops bis terve de die; but how agreeably was I furprized to find, in the courfe of a fortnight, her dyfpnœa had left her; the fight of her eye was perfectly reftored, without pain or difcharge; the feton healed up; and, in fhort, fhe was enabled to attend to her bufinefs, (a picker of wool) which fhe has continued to do without intermiffion to the prefent time, except, that about the latter end of the fummer fhe complained of a trifling cough and dyfpnœa, which was moft probably occafioned by the fudden tranfition obfervable at that time, from a warm and dry temperature to a cold and humid one: this was, however, immediately relieved, by having recourfe again to the tincture. It may be worthy of remark, that on the pain and dyfpnœa firft leaving her, an eryfipelatous inflammation, with a watery eruption, appeared round the whole furface of her lower extremities: On this occafion, I directed her to fprinkle fome powdered chalk fre-

 quently quently on the parts, which foon fubdued this unpleafant occurrence. Soon afterwards the catamenia began for the firft time to appear, and has continued ever fince at regular periods, though the difcharge is inconfiderable in quantity, and of a ferous appearance, being divefted of the florid colour it ufually affumes. I have thus given you a brief and imperfect account of a cafe, wherein I have enjoyed the felicity of refcuing a fellow-creature from undefcribable mifery, arifing from extreme bodily difeafe and the equally fevere pangs of wretched poverty. Her gratitude on this occafion is fuch as might be imagined, from one who is unexpectedly enabled to procure a humble though fufficient fubfiftence, independent of parochial affiftance, and not impelled by want to folicit the cold relief of cafual charity.

I fhall now advert to the advantage derived from its external application to old morbid ill-conditioned ulcers. One cafe only the prefent time will permit me to ftate.

A young girl, aged 13, of Box parifh in this county, more than a year fince, was fent to me with a large fcrophulous ulcer in the leg, of two years ftanding. I employed, for a confiderable time, every method in my power to heal it; but in vain I attempted to alter its unhealthy appearance, or promote granulation in any degree. I fteadily purfued for fome time Mr. Baynton's plan, with ftrips of adhefive plafter, and applied tight bandages, but with no effect. I frequently fcarified the furface of the ulcer, hoping, by exciting inflammation, to produce a difpofition to granulate. I fprinkled it alfo with pulv. rhabarb. and many other ftimulant fubftances, which only induced a confiderable degree of pain, without promoting in the leaft the benefit intended. Recollecting at laft that I had frequently heard of the good effects of oxygene applied to inveterate ulcers, and confidering the mufk to contain a portion from the combination of the nitric acid with the oil of amber, I determined to make trial of it; I therefore daily wafhed the ulcer with the tincture before defcribed, ftill continuing the ufe of the adhefive. I was foon happy in obferving effects the moft defirable; full and healthy granulations quickly formed; the ulcer affuming a florid, healthy appearance, healed gradually in the moft complete manner, and the new-formed cutis feems firm and compact, nor does there appear any probability of a relapfe. I am, &c.

*Bradford, Nov.* 20, 1800. O. W. BARTLEY, Surgeon.

